# Multidimensional Recording (MDR) and Data Sharing: An Ecological Open Research and Educational Platform for Neuroscience

**DOI:** 10.1371/journal.pone.0022561

**Published:** 2011-07-21

**Authors:** Yasuo Nagasaka, Kentaro Shimoda, Naotaka Fujii

**Affiliations:** 1 Laboratory for Adaptive Intelligence, BSI, RIKEN, Saitama, Japan; 2 Department of Neurological Surgery, Nihon University School of Medicine, Tokyo, Japan; Cajal Institute, Consejo Superior de Investigaciones Científicas, Spain

## Abstract

Primate neurophysiology has revealed various neural mechanisms at the single-cell level and population level. However, because recording techniques have not been updated for several decades, the types of experimental design that can be applied in the emerging field of social neuroscience are limited, in particular those involving interactions within a realistic social environment. To address these limitations and allow more freedom in experimental design to understand dynamic adaptive neural functions, multidimensional recording (MDR) was developed. MDR obtains behavioral, neural, eye position, and other biological data simultaneously by using integrated multiple recording systems. MDR gives a wide degree of freedom in experimental design because the level of behavioral restraint is adjustable depending on the experimental requirements while still maintaining the signal quality. The biggest advantage of MDR is that it can provide a stable neural signal at higher temporal resolution at the network level from multiple subjects for months, which no other method can provide. Conventional event-related analysis of MDR data shows results consistent with previous findings, whereas new methods of analysis can reveal network mechanisms that could not have been investigated previously. MDR data are now shared in the public server Neurotycho.org. These recording and sharing methods support an ecological system that is open to everyone and will be a valuable and powerful research/educational platform for understanding the dynamic mechanisms of neural networks.

## Introduction

How the brain achieves intelligence is one of the biggest unsolved issues. Many methods have been developed and used for revealing the mechanisms underlying brain functions. Electrophysiology in primates is one method. In the early 1960s, Evarts established [1] a method to allow chronic recording of single-cell activity as monkeys performed behavioral tasks. Since then, the method has been improved, and thousands of reports have been published. Various brain functions have been revealed by the technique, which has provided increasing detail about each function. To study the brain in detail, recent studies often use complex behavioral tasks that require a long training period (often over a year) before starting the neural recording. Such long-term training raises the question whether the neural mechanisms recorded under such task conditions reflect the original brain's functions or those developed through training. Of course, no one can answer this question unless we can track neural activity from the same neurons throughout the long training period, which is not yet possible because, in conventional single-cell recording, different set of neurons are picked up randomly at every recording session.

Another concern is the sparseness of temporal and spatial information obtained from single-cell activity. We can record the activity from usually less than 100 single cells from a few cortical and/or subcortical regions simultaneously. Each neural activity can be correlated to specific task parameters by aligning the activity to a specific task event and averaging over many trials. The proportion of neurons showing characteristic modulations is then calculated and compared between different brain regions. Temporal averaging and population analysis have been used to compensate for the sparseness in time and space. However, even with compensation, analyzing network dynamics of brain function using single-cell recording has limitations, and we need alternative methods to record and analyze the data.

Another concern about single-cell recording is the quality of the signal. Recent recording systems can isolate a single cell by the wave shape of each spike either offline or online. In most cases, sorting is done manually with subjective parameter setting. From the same data set, one researcher could isolate two neurons, but another might isolate four cells. This can cause problems when network analysis requires the precise timing of each firing. Several researchers [2], [3], [4] have tried to develop a method for automatic isolation to avoid subjective bias, but these automatic methods are not yet in common use because they require special electrodes using a bundle of thin wires, as a tetrode [5], [6], [7].

These concerns raised above are serious and a huge problem in neuroscience. However, they look minor when compared with the problem associated with the lack of social factors in the task environment [8], [9], [10]. So far, researchers have studied the neural mechanisms in a single brain isolated from social reality in which subjects are required to use social intelligence.

It is not easy to train a monkey to press a button when a light turns from green to red in an experimental room. It takes at least a few weeks to teach the simplest behavioral task. However, the same monkey that had trouble learning the simplest task can easily adjust its social behavior (e.g., whether taking food depending on the complex social context at a particular moment). In the natural social environment [11], the monkey has to consider many social factors such as its own intentions and those of others [12], past experiences with others, its hierarchical status [11], [13], [14], what others are paying attention to, and the relative distance between individuals [15]. The monkey has to consider the combination of these factors, called the social context, to perform a socially correct behavior. Any monkey can learn adaptive behavior through its development in society. We do not acquire our intelligence through learning in a button-pressing task, and the evolutional pressure of social adaptive behavior is an important part of our intelligence [16]. The social adaptive behavior is commonly observed [11] in most primates, and the neural mechanisms underlying learning functions in primates might provide a good path for understanding the mechanisms underlying human intelligence. However, conventional neurophysiology provides no clear answers because there are too many uncontrollable social parameters associated with reality. These uncontrollable parameters add uncertainty, which the conventional experimental design tries to avoid, but it is this uncertainty that the brain faces in reality.

We suggest that almost all previous single-cell recording results share these problems. To break this critical limitation to understanding realistic neural mechanisms, the multidimensional recording (MDR) system was developed [14], [17]. MDR comprises two major technical components: one for neural recording and the other for behavioral recording.

## Methods

### Neural recording in MDR

Many methods are used for in vivo neural recording. In developing MDR, we tested several recording methods and finally chose the electrocorticogram (ECoG) array for three reasons: the stability of the neural signal, the quantity of information, and the scalability.

The subdural ECoG array is used in epileptic patients [18] to identify the location of epileptic foci by implanting an array of electrodes in the subdural space. The array is normally left in subdural-space of patient for 2–3 weeks until the foci are located. During this period, the signal quality does not deteriorate, suggesting that this technique can be applied in neurophysiology to provide stable chronic recording. However, it required modification because the human ECoG was not designed for long-term use.

**Figure 1:**
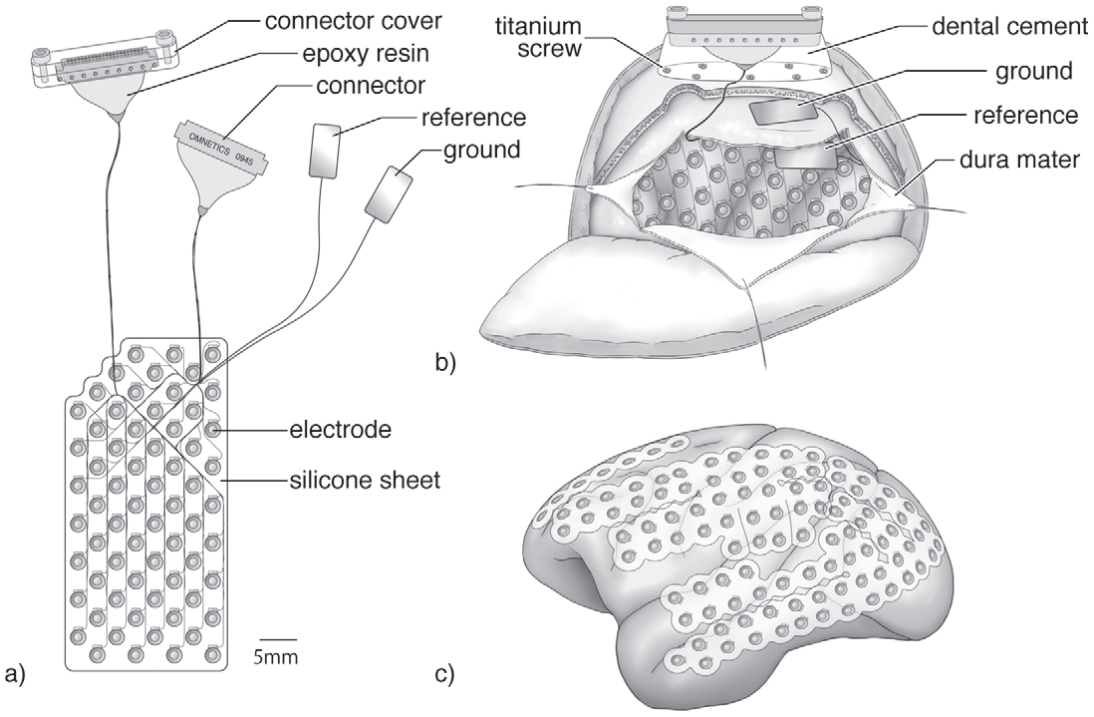
ECoG array, implantation and cortical coverage. a) Sixty-four channel ECoG array with connectors and the reference and ground electrodes. b) Schematic figure showing how and where the ECoG array, reference electrode, ground electrode, and connectors were implanted c) Schematic view showing how the 128-channel ECoG electrode can cover the entire lateral cortical surface.

To introduce subdural ECoG recording into monkey physiology, we modified a human ECoG array (Figure 1a and b) through two major modifications. The first was to place the reference and ground electrodes underneath the skull, whereas these are often placed on the skin surface in human ECoG recording. These reference and ground electrodes are made of rectangular platinum plate (4.5×9 mm), with one side insulated and the other side bare platinum. The reference electrode was placed between the silicone sheet of the subdural ECoG array and the dura mater with the platinum side facing the dura. The ground electrode was placed in between the dura and skull with the platinum side facing the skull (Figure 1b). The second modification was that we used a plastic connector (Omnetics connector corporation, Minnesota, USA) covered with a waterproof connector case, which was fixed securely on the skull with titanium screws and dental cement (Figure 1b). All experimental and surgical procedures were performed in accordance with protocols approved by RIKEN ethical committee (No. H22-2-202(4)) and the recommendations of the Weatherall report, “The use of non-human primates in research”.

At first, we implanted a 32-channel ECoG array in one monkey and found that the ECoG could provide a stable neural signal for several months [17]. However, evaluating the signal quality and stability was not easy because the signal and noise were not separable. We then applied a neural-decoding technique often used in the development of a brain–machine interface to see how much information was preserved in the ECoG signals and how long we could extract the same quality of information in the same manner. In our report, we made a prediction model of arm trajectory, which showed excellent prediction performance comparable with a prediction model based on single-cell activity [19]. The advantage of the ECoG array was the robustness of the prediction model with time. It turned out that one prediction model made with data obtained on one day could continue to give stable prediction performance for several months [17]. That had not been achieved before using a prediction model based on single-cell activity because of the low recording stability of single-cell recording with time. This finding suggested that the ECoG array could continue to detect the same properties and qualities of information essential for stable prediction. The stability is the biggest advantage of ECoG recording compared with other methods.

In a second animal, we almost doubled the density and number of electrodes (Figure 1a) to 64 and expected better decoding performance. However, the modification did not show significant improvement in decoding performance [17], which suggested that many electrodes are not always necessary if the electrodes are placed at the right place correctly.

We turned the ECoG design in the opposite direction. In a third animal, we again doubled the number of channels to 128 but with the same density as in the first trial. This modification meant that the ECoG could cover four times larger cortical area. Figure 1c indicates how the third-generation ECoG covered the cortex. The array could cover almost the entire lateral cortex from the occipital pole to the temporal and frontal poles continuously, including part of the medial wall. Rubehn and colleagues made similar progress [20], but there was no electrode in the medial wall in their study and no successful report about its long-term use.

The array gives us wider degrees of freedom in experimental design because it covers the visual, auditory, somatosensory, and motor areas, and the parietal and frontal association cortices. In conventional studies, especially when recording single-cell activity, the recording targets were limited to a tiny region; thus, the experimenter needed to design a specific task for each target region. For instance, if the neural mechanism underlying motor control was challenged, usually the experimenter never tried to place the electrode in the primary visual and auditory cortices although these regions are involved in all kinds of behavioral control. In contrast, researchers who are interested in visual or auditory processing never tried recording from the motor cortex even if these cortices are not separable in cognitive behavioral processing. One aspect missing in conventional studies, therefore, is that no brain region works independently; all brain regions are connected and work together. The ECoG array can cover wider areas that contribute in different ways to sensory and behavioral cognitive processing. This is the main advantage because we can capture the whole brain picture in one shot and do not need to combine separate pictures taken at different times.

Another advantage of the ECoG array is that it allows subjects more freedom in behavior during recording. We can record activity even in head-free conditions without losing the quality of the neural signal. This does not mean the recording has to be done with the head free, but researchers can choose any degree of freedom in the subject's behavior depending on their aims. Under any task conditions, the ECoG array would provide almost the same quality of neural information.

**Figure 2:**
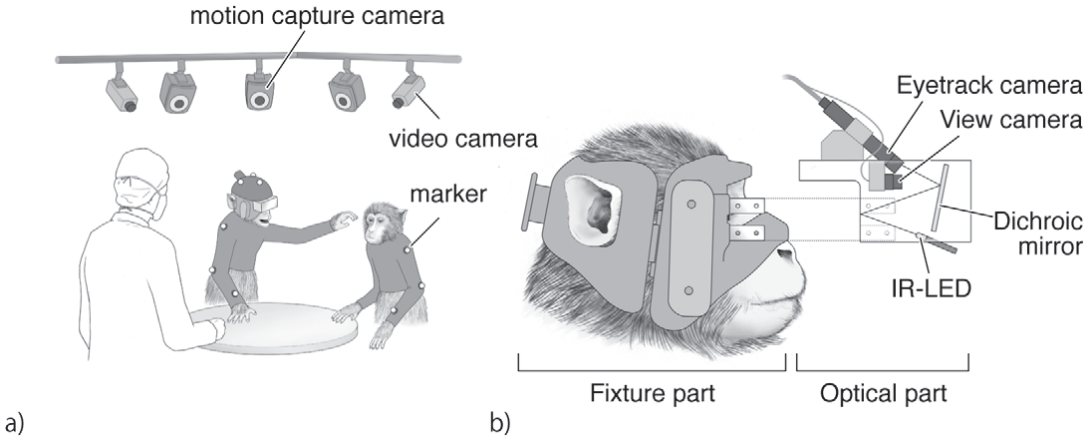
Experimental environment and eye tracking system. a) Sketch of the recording environment of MDR while the monkeys performed a social food-grab task. The monkeys wore an elastic motion capture suit tailored for each monkey. The monkeys sit on a primate chair with the lower body including the legs covered and restrained by a box and a collar fixed to a pole attached to the chair. Two monkeys were placed around a table. Reflective markers for motion capture were attached at the joints of both arms (shoulder, elbow and wrist) and the head. One monkey (left) whose ECoG activity was recorded wore a head-free eye-tracking system. Motion capture and video cameras were hung from the ceiling. b) Illustration of the head-free eye-tracking system. The eye-tracking system comprises two parts: a fixture part and an optical part. The fixture part was made of dental acrylic that fit the monkey's facial surface. The optical part had an infrared (IR) LED, dichroic mirror, eye-track camera, and view camera. The fixture part is tailored for each monkey and is attached to the optical part by screws.

In MDR, electroencephalogram (EEG) recording is occasionally made together with ECoG recording. In the case, EEG electrodes were integrated to a fixture part of eye-tracking system described below (Figure 2b).

### Behavioral recording in MDR

Although the ECoG array provides freedom in task design, the question arises as to how we should monitor and record the subject's behavior. One solution employed in MDR was to use an optical motion capture system [13], [14], [21] (Figure 2a). The motion capture system (Vicon Motion Systems, Vicon, Oxford, UK) detects reflective markers using multiple cameras and reconstructs the three-dimensional position of the markers with less than 1 mm resolution sampling at 120 Hz. In MDR, monkeys wear a custom-made elastic motion capture suit. Reflective markers are attached on the suit at multiple joints and on the head. The advantage of motion capture is that it does not restrain the subject's behavior, and the subject can move in any direction or manner. In our current setup, we use 10 motion cameras hanging from the ceiling, which can cover an area of almost 2×2 meters. Within the area, there is almost no limit of the number of markers and subject. For instance, when monkeys engaged in a social food-grab task, as shown in Figure 2a, there were two monkeys and one experimenter sitting in the area. The behavior of each of these monkeys and the experimenter was monitored by recording the motion of the markers. The location of each marker was reconstructed later.

In MDR, the eye position of free-behaving monkey was also recorded (Figure 2b). In human studies, many commercial products allow eye tracking in the head-free condition. However, in primate studies, no commercial products are available that allow head-free eye tracking. Eye position is very informative in social communication [22], because if one loses eye communication, essential joint attention is also lost. We developed a novel eye-tracking system (Figure 2b) for head-free monkey conditions (Takei Japan). The tracking system uses an infrared eye camera to detect corneal reflection. The goggle comprises two parts: an optical part and a fixture part. The fixture part is made of dental acrylic and is custom-made for each monkey to fit the facial curvature around the eyes. The optical part has a transparent shell with a dichroic mirror that reflects infrared light projected from IR-LED and has view camera, and eye camera integrated. The system can track eye position within 30 degrees. Two eye cameras can be installed in one goggle so that we can monitor both eyes and measure vergence to obtain depth information. These two cameras can be used for two subjects by splitting them for each. The sampling rate of eye tracking varies depending on the number of eye cameras used simultaneously. If two cameras are used, the sampling rate is 30 Hz for each camera. If only one camera is used, the sampling rate could be increased to 60 Hz. Pupil diameter can also be measured from the video image. By overlaying the eye position and view camera image, we can estimate which object the monkey is paying attention to in three-dimensional space.

Other environmental events can be monitored and recorded by multiple video cameras and a microphone hanging from the ceiling.

### Integration of multiple recording systems

**Figure 3:**
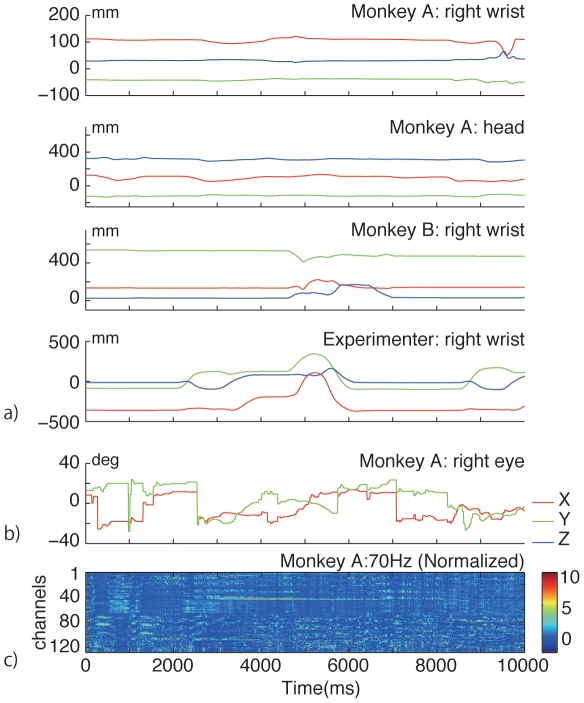
Simultaneously recorded motion capture data, eye tracking data and neural data for 10 sec. a) Three dimensional trajectories (X, Y and Z position indicated by red, green and blue lines respectively) of motion markers attached at Monkey A's right wrist, head, Monkey B's right wrist and Experimenter's right wrist. b) X (red) and Y (green) positions of eye tracking data of Monkey A's right eye. c) Normalized (z-score) power of neural data at 70 Hz recorded from 128 channel ECoG array.

In MDR, multiple recording systems are working during an experiment, and each recording system has its own sampling rate and time stamps. The aim of MDR is to capture all kinds of information simultaneously to see the causal relationship between internal events occurring in the brain and external events in the environment. The simplest solution for synchronizing multiple recording systems is to use a common external trigger that starts and stops all recording systems. In MDR, the Vicon motion capture system works as a recording hub because it can accommodate multimodal inputs at different sampling frequencies. The sampling rate is 120 Hz for motion capture data, 30 Hz for video input, and is flexible up to 1 kHz for 16-analog input. Figure 3 indicates motion data, eye track data and neural data recorded simultaneously at different sampling rate with MDR. Vicon system can also print Vicon's time stamp on video input and send out through an output port to other video devices. In our setting, the view camera image with overlaying eye position information is given to the Vicon video input and stored in the Vicon PC. At the same time, the same image is sent to a network video recorder (NVR-4016B: Nihon Brain Ware Co., Japan) with the Vicon time stamp printed. The video recorder stores the time-stamped images and the other video images captured from the environmental video cameras in parallel. Auditory information acquired from the microphone is also stored in the Vicon system as an audio file. Eye-tracking information is exported from the eye-tracking system to the Vicon analog input.

Neural data coming from the ECoG array is filtered and recorded by a Cerebus recording system (Blackrock Microsystems, Utah, USA). The system can accommodate 128 channels and is expandable by connecting multiple systems together. The ECoG data comprise a time series of analog data obtained from the array. EEG data is collected by NeuroPRAX system (eldith GmbH, Ilmenau, Germany). Neural recording and other recordings are triggered by an external TTL pulse, so that the starting and end points of the recording are synchronized.

### What can we do with MDR data?

MDR data are produced by the integration of multimodal time series signals, which have much higher dimensional data structure than conventional physiological data. For instance, in a classic physiological experiment, only a single spike train and sequence of task events can be recorded. Researchers align the spike train to the task event, average these over the trials, and calculate the significance of modulations in spike activity around the event time by comparing with the activity during a control period. The comparison is often made using simple statistical analysis. The method still works even when we can record multiple spike trains at once from multiple brain regions. It is also applicable to MDR data for studying event-related potentials.

As mentioned earlier, temporal information in the spike train is sparse, whereas ECoG activity provides a continuous time series of information in wide frequency bands, which is a characteristic advantage of measuring ECoG activity over single-cell activity. Figure 3c shows temporal series of power of neural signal at 70 Hz recorded from 128 channels. Another advantage of ECoG recording is that it has more spatial information in the brain network because it can cover wider brain regions. Technically, it is impossible to record single-cell activity from the entire cortex, but ECoG can (Figure 1c). Obtaining spatial information is not limited by spatial resolution but by the size of the area the electrodes can cover. ECoG has advantage in three domains—temporal, frequency, and spatial domains—which allow one to study different aspects of a neural network, e.g., synchronization, coherence, and causality between the same or different features of these domains.

Causality analysis has been applied recently in fMRI and EEG studies [23], [24], [25] to see functional network connectivity. However, ECoG activity is much more suitable for this analysis because of the greater temporal and spatial resolution. Causality analysis can measure information flow between brain regions. The difference between conventional cross-correlation and causality analysis is that the latter provides information about the direction of the flow. Once information flow and the strength of the connection between regions are described by causality analysis, we can draw functional diagrams about how the information is circulating in the neural network.

MDR collects not only neural data but behavioral and environmental data as well. In conventional experiments, all events are designed carefully before the experiment starts, and one cannot analyze neural activity related to events that were not planned in the original experimental design. In contrast, in MDR within the natural social environment, we can extract a variety of additional events that were not planned originally. These planned and post hoc events are equally useful for later detailed analysis. Especially in electrophysiology, experimental results tend to differ little from what one expects, and the researcher often needs additional experiments under different task conditions. However, in MDR, the additional task conditions might be found, and can be extracted, by filtering behavioral and environmental information. This is another advantage of MDR that conventional methods lack.

### Sharing service: Neurotycho.org

How to share experimental data is a hot topic in science. Neuroinformatics [26], [27] is a research field that provides a data-sharing service in neuroscience. Only a few services provide neural information that is useful in a practical sense for revealing new information about neural mechanisms, despite constant requests from those working on the theoretical aspects [28].

Although everyone agrees that sharing data is “good”, the reality is that it has not yet become a major trend. This raises the question why researchers do not wish to share their data. There are two possible reasons. One is the concern about copyright issues. People tend to prefer to retain copyright of their data. If experimenter A uses experimenter B's data through data sharing and wants to publish new findings, B naturally thinks that B's work should be cited in the paper as the original owner of the data or that B should be a coauthor. However, this is not guaranteed unless A and B agree on this issue before sharing the data. The process of formulating an agreement and following copyright control requires effort by each researcher, which can reduce the perceived benefits in comparison with the effort. This may explain why people are reluctant to share data.

Another reason is the more technical issue relating to the usability of the service, which is tightly linked to the issue mentioned above. Engineers who develop a data-sharing service are concerned about access control, although users may not be aware of the need for and complexity involved in such control. The access policy can become complex because the system has to be able to accommodate many different cases while protecting the data set against illegal access and ensuring the system's security. Without the security, no one will want to keep data sets on the server. Security and usability thus become a trade-off; that is, the database server with robust security is not necessarily user-friendly to the owners and users of the data because the complex settings needed to ensure access control become burdensome. Such complexity often deters people from using the service. This is a dilemma faced by those involved in designing a data-sharing service.

On the other hand, if we are not concerned about copyright and security issues, the dilemma will disappear because all of the data set on the service is open to the public and there is no need for strict access control. Our data-sharing service Neurotycho.org was designed based on this idea. All data on Neurotycho.org are available to everyone. Users can download MDR files at no cost. Users first register a valid e-mail address for use, and once the user's account is generated, he or she can download and use files without any restrictions because the data set is distributed as zero copyright (CC0) under Creative Commons licensing [29]. A valid e-mail address is not used to confirm the user's identity and is used only for communication purposes between users and us.

So far, more than 4 Gbytes of data have been uploaded on our server, and more than 350 researchers have registered as users in seven months.

## Discussion

### Advantages and disadvantages of MDR

MDR can collect a wider range of information than conventional methods. The strongest advantage of MDR is that all of the data from different modalities are collected at the same time. One criticism is that MDR is collecting a pile of unnecessary information without controlling those parameters that should be controlled. We have a different view. In conventional physiological methods, some claim that most behavioral and environmental parameters are controlled. However, we believe that this is not always true because most parameters that are assumed to be controlled are not controlled as the researchers expected at the start of the experiment. For instance, when the head is fixed, there is an assumption that there is no muscle activity related to head motion. However, monitoring muscle activity in the neck sometimes shows muscle activity in the neck during the task because the monkey can move its body even with the head fixed. The problem is that the researcher often ignores this kind of possibility simply because it is accepted as the standard method. Many other assumptions that are also accepted in standard conventional methods in terms of behavioral control are being challenged by MDR. Instead of ignoring certain types of activities, in MDR we decided to monitor all kinds of parameters that we could record. MDR does not ignore any possibility. Of course, we cannot monitor everything, but the principle in MDR is that we will collect as much data as possible to challenge various possibilities because monitoring is better than ignoring.

It should be emphasized that MDR is not just an experimental technique for experiments only in social neuroscience; MDR can be an ideal and common research platform for any type of physiological experiment. In MDR, behavioral restrictions can be adjusted depending on the experimental goals. We can modulate the restriction level gradually from the level of conventional methods to restriction-free methods while recording neural activity continuously. This means that MDR may help bridge between conventional results and new results obtained under restraint-free natural social conditions. MDR will provide a new view of neural mechanisms based on previous findings.

### Ecological platform for neuroscience

Besides developing the MDR technique, we launched a data-sharing service called Neurotycho.org. Neurotycho was named after Tycho Brahe, who was a mentor of Kepler. Kepler would not have been able to confirm Kepler's law of planetary motion without Brahe's huge data set. We hope that sharing our data set in the public domain will follow Kepler's lead and advance the field of neuroscience.

Data sets on Neurotycho.org contain data whose analysis is complete as well as data that have not been analyzed yet. In preliminary studies, we recorded MDR data, but not all data have been analyzed thoroughly. Such data sets may not be useful in our studies but may be useful in other aspects. Thus, we decided to put them on the server.

**Figure 4:**
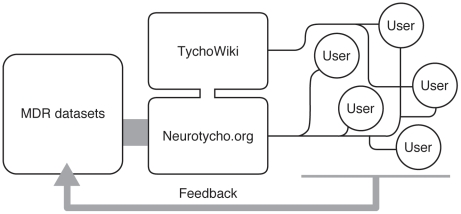
Neurotycho and open research platform. The combination of MDR and Neurotycho.org will provide an open platform for research and education in neuroscience. The interaction between the users and us will improve the quality of the service.

Neurotycho is not simply offering data sets to users. We are trying to be more interactive with users (Figure 4). For instance, we uploaded one auditory task data set upon request from one user. We are willing to share our data recorded in the past when a user requests such data.

If a user requests a type of data set recorded under specific conditions that we did not record, we are happy to record the new data set for them if the question the user wants to address through the data is scientifically valid. In this case, we will ask the user to complete a collaboration agreement before recording because we need this to make an additional allocation of budgetary and human resources in our laboratory.

Data sets become more useful when additional information is combined. Neurotycho Wiki was developed for this purpose. We are currently trying to upload various texts related to the server contents—MATLAB codes, annotation of tasks, surgical methods, etc. For beginners, we are writing instructions for setting the analysis environment using Python, open source software. This may help younger students to start learning about neural mechanisms using raw neural and behavioral information that they could not access otherwise.

Before MDR, it was not easy to obtain high-dimensional physiological data. This is still true in most laboratories because of the setup costs. However, once the setup is complete, data collection becomes relatively inexpensive. This is the greatest virtue of MDR that pushed us to start data sharing.

MDR and Neurotycho.org are an ideal combination for circulating physiological data sets and furthering ideas about understanding neural mechanisms. The combination will help build an ecological neuroscientific community that will accelerate the understanding of complex brain functions.
